# Planning of surgical activity in the COVID-19 era: A proposal for a step toward a possible healthcare organization

**DOI:** 10.1177/0391560320938579

**Published:** 2020-07-15

**Authors:** Bernardo Rocco, Alessandra Bagni, Elisabtta Bertellini, Maria Chiara Sighinolfi

**Affiliations:** 1 University of Modena and Reggio Emilia, Modena, Italy; 2 Department of Anesthesiology, Aienda Ospedaliero Universitaria of modena, Italy; 3 Azienda Ospedaliero-Universitaria di Modena, Modena, Italy

**Keywords:** Surgery, pre-admission pathway, COVID-19 transmission, restoration, urology

## Abstract

Health-care systems worldwide are experiencing a decline in elective surgical activity during the current COVID-19 pandemics. The progression of morbid conditions—especially of cancer—and the uncontained increase of waiting list for scheduled interventions are the major drawbacks. We propose a possible organization of a COVID-19 free hospital or hub, that include both patients’ and workforce’s preparation before entering the facility. The addition of a planned pathway for the whole workforce (physicians, nurses, cleaning and transporting crews, etc.) represents the basis of the program, and involves COVID-19 testing and subsequent self-isolation before entering the hospital, avoidance of work in non-COVID free areas, a strategic fractioning with a multilayer coverage system of care, periodic re-testing. Based on these suggestions, the realization of a COVID-19 free hospital could be achieved, allowing the continuation of a safe surgical activity in view of a possible restoration of non-urgent activity.

Health care systems worldwide are currently facing the COVID-19 pandemic. In the countries mostly hit by its dramatic outbreak, such as Italy, the health care delivery has been abruptly redistributed to allocate human resources, beds, operating room (OR) spaces and intensive care units (ICUs) to the care of symptomatic or critically ill patients.^1^

As a result, the volume of elective procedures dramatically dropped: this occurrence applied either to diagnostics, such as gastro-intestinal, and to scheduled surgical interventions.^1,2,3^

As surgeons at an academic hospital in Northern Italy (Modena, more than 180.000 inhabitants and 1100 bed availability), we experienced a sudden—but inexorable—decline in surgical activity on a week-by-week basis, by the beginning of the epidemic in Italy, 22 February. A similar trend is extending progressively to other cities and regions, due to the need for an extra-care availability for COVID-19 patients.^3^

The first response to virus spreads consisted of measures to contain the infection while attempting to preserve the routinary health-care activity, that is, dedicated triage at the emergency room (ER) units, protection gears for patients and personnel, creation of COVID-19-dedicated areas.^1,2^

However, the lesson we quickly learned is that virus containment within a single institution is difficult or impossible to be achieved:^2^ hospitals are quickly overwhelmed by the number of infected patients, and the risk of transmission inside the facility is extremely high.^4^

From the Wuhan experience, nosocomial contamination is responsible for 41% of the cases, with caregivers being exposed to symptomatic infections or, opposite, being vectors of transmission as well.^5^

The risk of COVID-19 in fragile patients elected to surgery is a concern that surgeon may be responsible of, and, regrettably, end up on trial for. The mortality rate of patients having their planned surgery during the incubation period of COVID-19 is estimated to be as high as 20.5%.^6^

This concern obviously applies to oncologic patients: an active neoplastic condition is a risk factor for a fatal evolution of COVID-19 disease, detected in 20% of COVID-19-related deaths (Source: ISS, Italy).

Furthermore, the possible development of post-operative respiratory distress can compromise the eligibility of oncologic patients to adjunctive therapies, such as chemo, immunotherapeutics or radiation treatments, thus impairing the execution—and ultimate, the efficacy—of a combined treatment modality. How to balance the risk of COVID-19 nosocomial transmission with the inexorable progression of untreated conditions, life-threatening as well? No certain instructions are available yet.^7^

The surgical community responded with the immediate draft of “prioritization charts” and COVID-19 adapted guidelines, cross-sectioning all surgical specialties: a chart arranges a sort of to-do-list, stating priority and timing of each procedure—while the emergency is going on.^8,9^

But the time length of the COVID-19 emergency is something we are not aware of and the evolution of the pandemic is still uncertain: meanwhile, the provision of surgery should continue to be an essential aspect of healthcare systems.

The creation of COVID-19 free (C19-free) hubs or facilities could be the key-point to deliver a continuous and safe care to patients elected to surgery. To this effort, the pre-admission period seems to be the key point and, at the same time, the point of weakness of the process.

Flow charts displaying the ideal pre-admission can be adopted from oncology: the key steps include a visit to confirm the indication to treatment (with consideration of alternatives), the triage for COVID-19 symptoms at a preliminary visit, the re-triage for symptoms at the moment of hospitalization, 2 weeks thereafter.^10^

Simonato et al.^11^ described another ideal pathway to obtain a C19-free hospital: it includes the nasopharyngeal swab in the pre-admission phase and, if negative, patient’s isolation inside the facility, in a single room of the hospital ward, up to surgical intervention. Despite the brilliant suggestion, its feasibility inside most of the Italian facilities could be a matter of argue. Besides, beyond patients’ testing and strict isolation, the workforce too cannot afford not to follow similar stringent rules.

Actually, the challenge posed by COVID-19 are unique and different from previous epidemics (including the 2003 SARS);^12,13^ COVID-19 accounts for:

- A variable incubation period (2–14 days)
- Early onset of infectious period (COVID-19 transmission begins during the early phase of illness, with viral peaks while the patient is asymptomatic); presymptomatic transmission also makes basal screening (ie temperature) uneffective^14^
- A wide time-lapse (2–5 days) between the onset of symptoms and peoples’ seeking medical attention, that enhances the community transmission^14^
- Nasopharingeal swabs (with RT-PCR to detect viral RNA, the current gold standard) have limitations such as short detection windows, false sampling, cross-contamination of samples, inconsistence of sample collections and preparations.^14^
- Laboratory detections and radiographic images are not always in agreement with clinical features of COVID-19.
- Serologic testing for COVID-19 are still prone to variability in false-negative reporting; they rely on the timing of a detectable immune response, that could vary; serologic testing also miss infections among immunocompromised people not producing antibodies^15^

The aforementioned issues—that limit a prompt detection of COVID-19 cases—allow pathogens transmission also inside a supposed C19-free hub or facility, through asymptomatic subjects that could be either patients and health-care workers, the latter acquiring infection at a community level or while working in non- C19-free areas in mixed hospitals. Therefore, the realization of a complete C19-free facility should be planned involving either patients and workforce (Figure 1).

**Figure 1.**
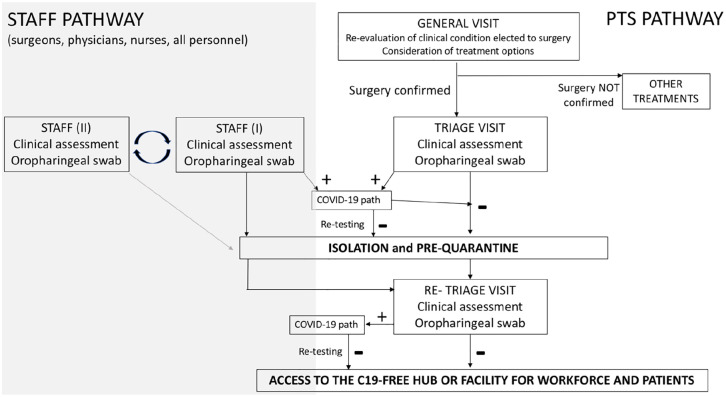
Proposal for a C19-free facility of hub.

From the workforce point of view, a strategic ractioning with a multilayer coverage system of care could be suggested at either surgeons’ and nurses’ level. It translates into the adoption of a pre-quarantine of the staff (or of part of the staff, to allow a rotation), plus oropharyngeal swabs before entering a C19-free facility. This rule should apply to all caregivers, including physicians, nurses, cleaning and transporting crews, and so on, for whom the work in non C19-free facilities is forbidden in the prior 2 weeks. Periodic re-testing is needed to verify the absence of asymptomatic infection in caregivers.

Similarly, patients should be invited to adhere a strict protocol, that includes a pre-quarantine period (with complete isolation from external environment), followed by a triage visit and nasopharyngeal swab at the moment of hospitalization.

The matter of patients’ compliance to the pre-isolation restriction could arise; however, we believe that a detailed explanation of the post-operative risk connected to COVID-19 could be a mischief toward harmful behaviors. In this setting, an informed consent structured to include infectious risk is mandatory and already planned by several institutions.

The realization of a C19-free facility could be a challenging matter, prone to arguments and criticisms; furthermore, whether this proposal could be effective, it is still debatable.

In the meanwhile, we have to keep in mind the definite role of surgery, that is the result of decades of scientific researches and technological improvements. Surgery is invoked as the gold standard treatment of most of the locally confined tumors, as a key point inside multimodal approaches, surgery is often the only cure of non-malignant conditions that, if left untreated, could become life-threatening as well.

To avoid the “risk of a recession” of the whole health-care provision—screening, diagnostics, treatment, and surgery too—and to restore elective activity, we have to rearrange promptly our health system and to reach a consensus on the way to do so.

## References

[1] Grasselli G Pesenti A Cecconi M Critical care utilization for the COVID-19 outbreak in Lombardy, Italy. JAMA 2020; 323: 1545–1546. 32167538 10.1001/jama.2020.4031

[2] Rosembaum L Facing COVID—19 in Italy—ethics, logistics, and therapeutics on the epidemic’s front line. N Engl J Med 2020; 382: 1873–1875. 32187459 10.1056/NEJMp2005492

[3] Rocco B Sighinolfi MC Sandri M, et al The dramatic COVID-19 outbreak in Italy is responsible of a huge drop in urological surgical activity: a multicenter observational study. BJU Int. Epub ahead of print 18 June 2020. DOI: 10.1111/bju.15149. PMC7322984 32558053

[4] Sighinolfi MC Rocco B Mussini C COVID-19: importance of the awareness of the clinical syndrome by urologists. Eur Urol. Epub ahead of print 25 4 2020 DOI: 10.1016/j.eururo.2020.03.029. PMC7176388 32345522

[5] Wang D Hu B Hu C, et al Clinical characteristics of 138 hospitalized patients with 2019 novel coronavirus—infected pneumonia in Wuhan, China. JAMA 2020; 323(11): 1061–1069. 32031570 10.1001/jama.2020.1585 PMC7042881

[6] Lei S Jang F Su W, et al Clinical characteristics and outcomes of patients undergoing surgeries during the incubation period of COVID-19 infection. Lancet 2020; 21: 100331. 10.1016/j.eclinm.2020.100331 PMC7128617 32292899

[7] Kashi AH COVID-19, urologists and hospitals. Urol J 2020; 17: 327. 32207541 10.22037/uj.v0i0.6064

[8] EAU Guideline Office Rapid Reaction Group: an organization—wide collaborative effort to adapt the EAU guidelines recommendations to the Covid-19 era, 2020, https://uroweb.org/guideline/covid-19-recommendations/

[9] Ficarra V Novara G Abrate A, et al Urology practice during COVID-19 pandemic. Minerva Urol Nefrol. Epub ahead of print 23 3 2020 DOI: 10.23736/S0393-2249.20.03846. 32202401

[10] Ueda M Martins R Hendrie PC, et al Managing cancer care during the COVID-19 pandemic: agility and collaboration toward a common goal. J Natl Compr Canc Netw 2020; 18: 1–4. 32197238 10.6004/jnccn.2020.7560

[11] Simonato A Giannarini G Abrate A, et al Pathways for urology patients during the COVID-19 pandemic. Minerva Urol Nefrol. Epub ahead of print 30 3 2020 DOI: 10.23736/S0393-2249.20.03861. 32225135

[12] Wang Y Wang Y Chen Y, et al Unique epidemiological and clinical features of the emerging 2019 novel coronavirus pneumonia (COVID-19) implicate special control measures. JAMA 2020; 92: 568–576. 10.1002/jmv.25748 PMC7228347 32134116

[13] Wilder-Smith A Chiew CJ Lee V Can we contain the COVID-19 outbreak with the same measures as for SARS? Lancet 2020; 20: 102–107. 32145768 10.1016/S1473-3099(20)30129-8 PMC7102636

[14] Med Q Guan X Wu P Early transmission dynamics in Wuhan, China, of novel coronavirus—infected pneumonia. N Engl J Med 2020; 382: 1199–1207. 31995857 10.1056/NEJMoa2001316 PMC7121484

[15] Abbasi J The promise and peril of antibody testing for COVID-19. JAMA 2020; 323: 1881–1883. 32301958 10.1001/jama.2020.6170

